# 15-year survivorship of a unique dual-modular femoral stem in primary hip arthroplasty

**DOI:** 10.1186/s12891-024-07422-0

**Published:** 2024-04-22

**Authors:** David F. Scott, Kade Eppich, Edin Mehić, Celeste Gray, Crystal Lederhos Smith, Michael Johnston

**Affiliations:** 1 Elson S. Floyd College of Medicine at Washington State University, 412 E Spokane Falls, Blvd, Spokane, WA 99202 USA; 2 Spokane Joint Replacement Center, Inc., Spokane, WA USA; 3 University of Washington, School of Medicine, Seattle, WA USA

**Keywords:** Total hip arthroplasty, Modular femoral stem, Dual-modular, Dislocation, Leg length discrepancy, Implant survivorship

## Abstract

**Background:**

Hip offset, version, and length are interdependent femoral variables which determine stability and leg length. Balancing these competing variables remains a core challenge in hip arthroplasty. The potential benefits of modular femoral stems have been overshadowed by higher rates of failure. The objective of this study was to assess the survivorship of a unique dual-modular femoral stem at an average 15-year follow-up period.

**Methods:**

The records of all patients with osteoarthritis who underwent primary total hip arthroplasty with this device between 2004–2009 were reviewed. There were no exclusions for BMI or other factors. We examined the data with Kaplan–Meier survival analysis. The primary endpoint for survival was mechanical failure of the modular neck-body junction.

**Results:**

The survivorship of this device in 172 subjects was 100% with none experiencing mechanical failure of the modular junction at an average of 15 years. 60 patients died of causes unrelated to their THA and 9 patients were lost to follow-up. There were three early (≤ 12 months) dislocations (1.7%), and seven total dislocations (4.1%). 16 patients underwent reoperations during the follow-up period, none for any complication of the modular junction. Radiographic results showed well-fixed femoral stems in all cases. There were no leg length discrepancies of greater than 10 mm, and 85% were within 5 mm.

**Conclusion:**

There were no mechanical failures of the modular junction in any of the subjects over the average 15-year period, demonstrating that this dual-modular design is not associated with increased failure rates. We achieved a 1.7% early dislocation rate and a 4.1% total dislocation rate without any clinically significant leg length discrepancies.

## Introduction

Total hip arthroplasty (THA) is a commonly performed orthopaedic procedure. Primary THA volume in the US is projected to grow by 71% to 635,000 procedures annually by the year 2030 [1], and another report estimates 850,000 THA/year in 2030, and 1.43 million THA procedures annually by 2040 [2].

Hip offset, version, and length are interdependent variables which determine stability and leg length. Selection of the best offset is critical to successful THA, to ensure stability and appropriate abductor function, yet this can present clinical challenges in those cases in which the degree of offset desired for stability causes an increased leg length. Balancing these competing variables remains a core challenge in hip arthroplasty [3] and accurate restoration of leg length and dynamic stability are essential to a successful THA [4].

Monoblock stems with modular femoral heads are limited in their ability to adjust offset and length compared to stems with additional modularity [5]. Modular, multi-piece femoral stems were introduced in the 1960’s in an effort to improve the anatomic restoration of hip offset and leg length. Some stems that possess modularity between the neck and proximal body (dual-modular) may allow fully independent adjustments in leg length and lateral offset, though many of these designs do not allow independent adjustment of these variables. Some dual-modular designs also allow adjustment of version.

Currently, the potential benefits of dual-modular femoral stems have been overshadowed by higher rates of failure compared to traditional devices, which is generally found to be around 5% [6–14]. The dual-modular exchangeable-neck devices have demonstrated particularly poor results [12, 15–23]. However, some studies have shown promising results with other modular stem designs [24–30].

The senior author has used a unique dual-modular stem design for two decades with good clinical results. The objective of this study was to assess the survivorship of a dual-modular femoral stem possessing a unique design at an average 15-year follow-up period, with mechanical failure of the modular neck-body junction the primary endpoint. We hypothesized that the implant survivorship of this modular stem was comparable to the survivorship of clinically successful non-modular femoral stems at similar follow-up periods.

## Patients and methods

Following Institutional Review Board approval, we performed an implant survivorship analysis utilizing the senior author’s institutional database and available electronic medical records. All cases were carefully planned preoperatively using anteroposterior pelvis x-rays and manual templating with selection of the most appropriate stem size for filling the femoral canal, and then the most appropriate modular neck that best reproduced the patient's normal offset and leg length. All procedures were primary THAs performed by the senior author using a standard posterior approach without posterior repair. Intraoperatively, leg lengths were measured clinically by comparing the relative position of the bent knee compared to the opposite extremity. All patients followed a standardized postoperative rehabilitation protocol allowing immediate full weight-bearing.

The study population consisted of 172 patients who underwent primary THA with a second-generation modular femoral stem between 2004 to 2009, providing an average of 15 years of follow-up data with analysis ending in December 2021. Indications for THA were primary degenerative arthritis (166), osteonecrosis/ischemic necrosis (5), and developmental dysplasia of the hip (1); revisions, inflammatory arthritis, and fractures were excluded. Using our research database and records, we identified 381 patients with these diagnoses who received a primary cementless hip replacement between 2004 and 2009. Of the 381 paitents, 172 patients received the study stem. During this time, our institution was involved in research protocols which specified other devices, thus a subset of all primary THA candiates received the study device. Otherwise, there was no exclusion for BMI or other factors such as diabetes or smoking history. We reviewed medical histories, databases, and contacted patients as necessary to determine their study implant status. We reviewed all available supine pelvis x-rays and performed magnification-corrected measurements of leg length before and after surgery. We collected information related to adverse events, implant survival and revision surgery, and any complications associated with the index THA.

### Implants

**Fig. 1:**
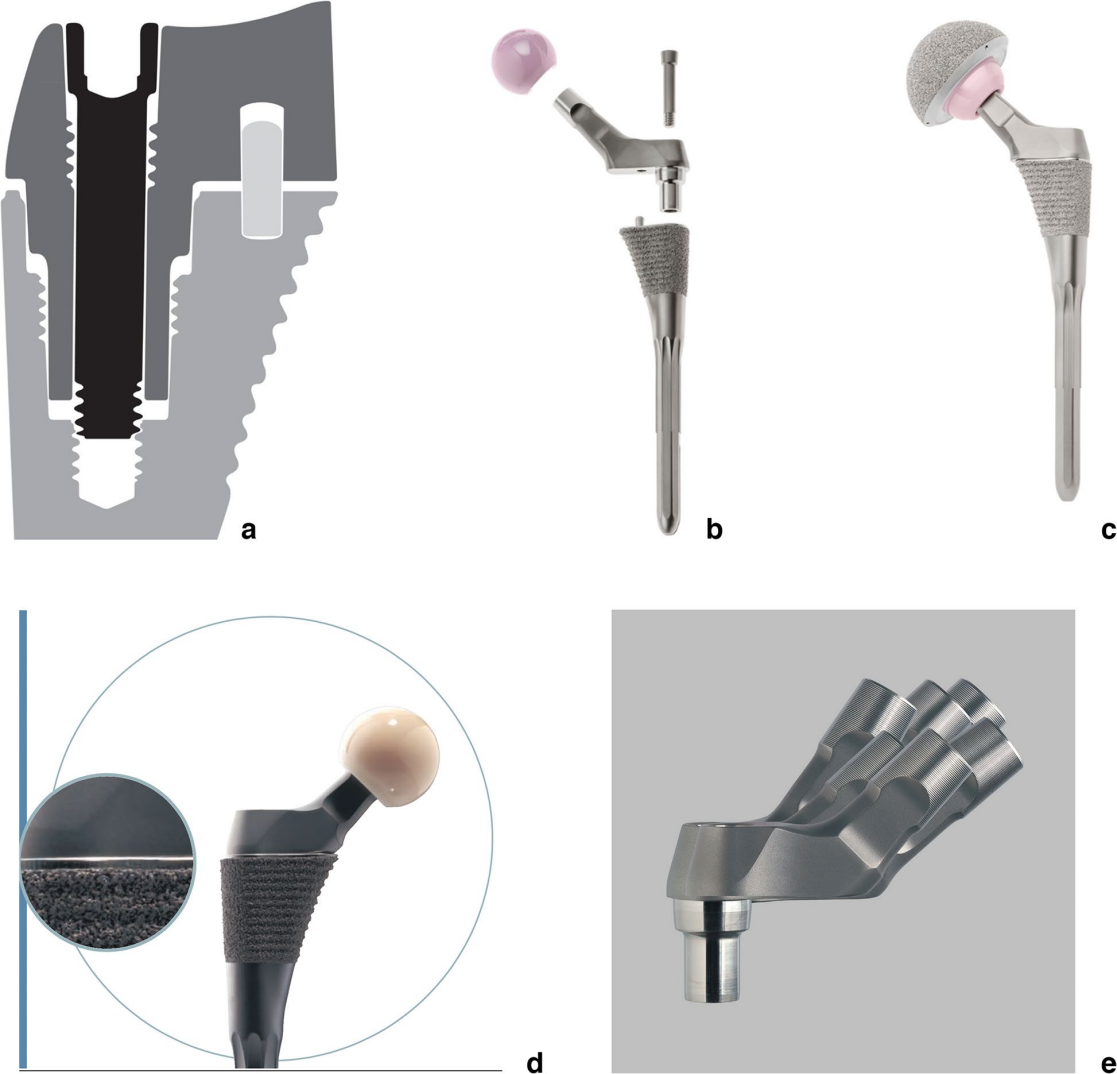
**a** Illustration of the dual-press mechanism, showing the proximal body, modular neck, locking bolt, and anti-rotational pin. **b** and **c** This figure depicts “exploded” and “assembled” views of the two modular femoral implant components. **d** This close-up photograph of the assembled implant shows that the neck and stem have no gap, converting into a monoblock construct similar to non-modular designs. **e** This illustration depicts some of the multiple neck options available for any stem

**Fig. 2:**
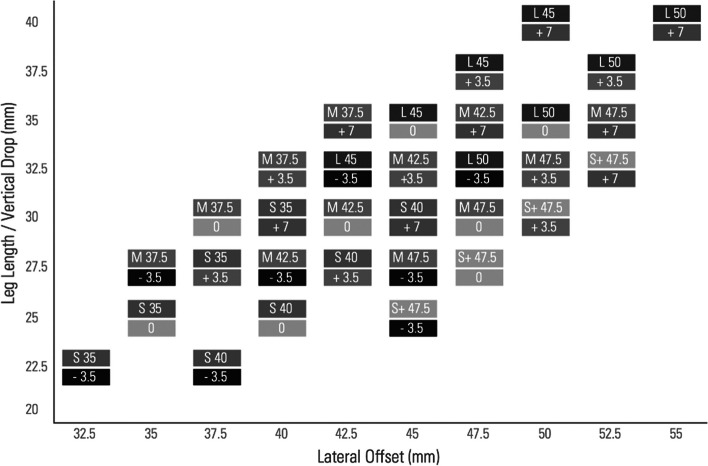
The illustration depicts the range of offset/length options available with this modular femoral system

The study femoral implant was the Omni Apex Modular™ second-generation femoral stem (Corin USA, Raynham, MA). The body and modular neck is made of titanium alloy and has a proximal titanium plasma sprayed surface. The anti-rotational pin is cobalt chromium alloy, which was changed from a diameter of 3.175 mm (first generation) to 4.775 mm to make it more robust and resistant to failure. It has a “fit and fill” design with a proximal-filling body and a cylindrical, splined, and split distal stem, implanted with a “ream and broach” surgical technique. The stem utilizes a modular “dual-press” connection mechanism (Fig. 1a) that seats the neck fully against the proximal surface of the body of the stem, significantly decreasing the likelihood of a gap. This subsequent elimination of micromotion, creates a final construct similar to monoblock designs (Fig. 1b, c, and d) [29, 31, 32]. The modular design of the neck fully uncouples the variables of lateral offset and vertical height, providing a number of options (Fig. 1e). Version angles, and head size are also adjusted independently. There is an offset/length chart that provides a match for any contemplated anatomic variance. (Fig. 2) Any stem body can be assembled with any modular neck option, with the exception that a neck with an offset greater than 45 mm should not be coupled with the two smallest stem sizes.

The acetabular components utilized were all press-fit titanium shells, with 12 of the 172 subjects receiving modular polyethylene liners and cobalt-chromium femoral heads with a diameter of either 28 mm or 32 mm, and the remaining 160 subjects receiving large-diameter metal-on-metal (MOM) articulations.

### Statistical analysis

Kaplan–Meier survivorship analysis was performed; end points were defined as (1) mechanical failure of the dual-modular junction and (2) revision surgery for failulre due to all reasons except MOM/ATLR. Patients who were deceased or lost to follow-up were censored. All data are presented as mean ± standard deviation.

## Results

The 172 patients included 71 men and 101 women with a mean age of 65 years (range, 33 – 94 years) at the time of surgery. 60 patients died of causes unrelated to their THA with their implant intact and 9 were lost to follow-up. Their mean body mass index (BMI) was 30.1 kg/M^2^ ± 5.8 kg/M^2^, with specific groups described in Table 1.

**Table 1:** Patient body mass index

Number of Patients	BMI (kg/M^2^)
37 (22%)	< 25
61 (35%)	25—29
44 (26%)	30—34
19 (11%)	35—39
11 (6%)	40—45

Of the 60 patients who died of causes unrelated to their THA, the average time to death was 7.2 ± 3.5 years post-THA (range, 0 – 10 years), and average age at death was 81. The nine patients who were lost to follow-up were followed for 9, 12, 16, 22, 35, 42, 44, 51, and 54 months with an average of 30 months. Emails, phone calls and letters were utilized to attempt to reconnect with subjects that were lost to follow up. Of the 60 that died and 9 that were lost to follow-up, none had experienced failure of their modular femoral stem up to the censure date.

There were 16 patients who underwent reoperations during the follow-up period. Ten subjects were revised at an average of 6.4 years post op for complications of large-diameter metal-on-metal articulations consisting of trunnionosis of the large-diameter cobalt-chromium head-titanium modular neck junction, elevated serum metal ion levels, and pseudotumor with adverse local tissue reaction (ALTR). Four of these MOM failures also had instability. There was one additional instability case which was not a large-diameter MOM case. The five instability cases were performed at 11 months, 2 years, 3 years, 7 years, and 8 years post-op; one subject had a 32 mm head, and the other four had large diameter MOM articulations (diameters 44, 46, 46, and 48 mm); Three subjects were revised for late joint infection (1 year, 3 years, and 8 years post-op) and two for acetabular loosening (Table 2).

**Table 2:** Reoperation diagnoses

10	MOM/ALTR
3	Chronic Infection
2	Aseptic loosening-Acetabulum
1	Instability alone
**16**	**Total Reoperations**

The femoral stem was removed in the three infection cases. All of the cases of MOM/ALTR dual-modular junctions were disassembled and then reassembled with a new modular neck. In the single instability case not associated with a failed large diameter MOM articulation, the modular neck component was exchanged for one with different version, offset and/or length; in all cases there was no further instability after the revision. In all 11 cases where the modular neck-stem junction was disassembled (10 MOM/ALTR, one isolated instability), the modular neck-stem junction was examined visually and found to be absent of any visible corrosion or any gross evidence of metallurgical failure or fracture. The femoral stem was retained in all 13 non-infection cases. Two other patients experienced a dislocation postoperatively, each before one year postop, treated with a closed reduction, and experienced no further instability.

**Fig. 3:**
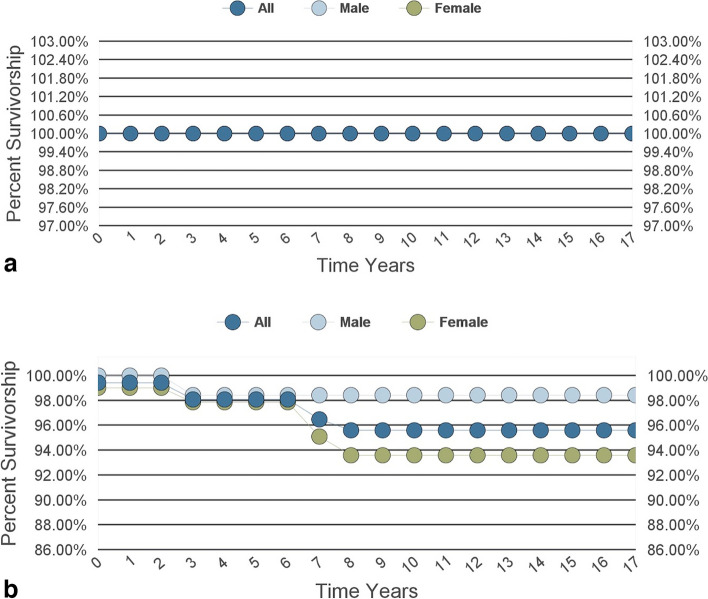
**a** Survivorship curves with mechanical failure of the modular junction as endpoint. **b** Survivorship curves with all failure reasons except MOM/ALTR

We found no mechanical failures of any kind, including breakage or corrosion, of the modular neck-stem junction at the mean follow-up period of 15 years (range, 13.2—18.1). Statistical analysis of the data found the Kaplan–Meier survivorship with mechanical failure as the endpoint was 100% (Fig. 3a) and all reasons except for MOM/ALTR failures was just under 96% (Fig. 3b).

**Fig. 4:**
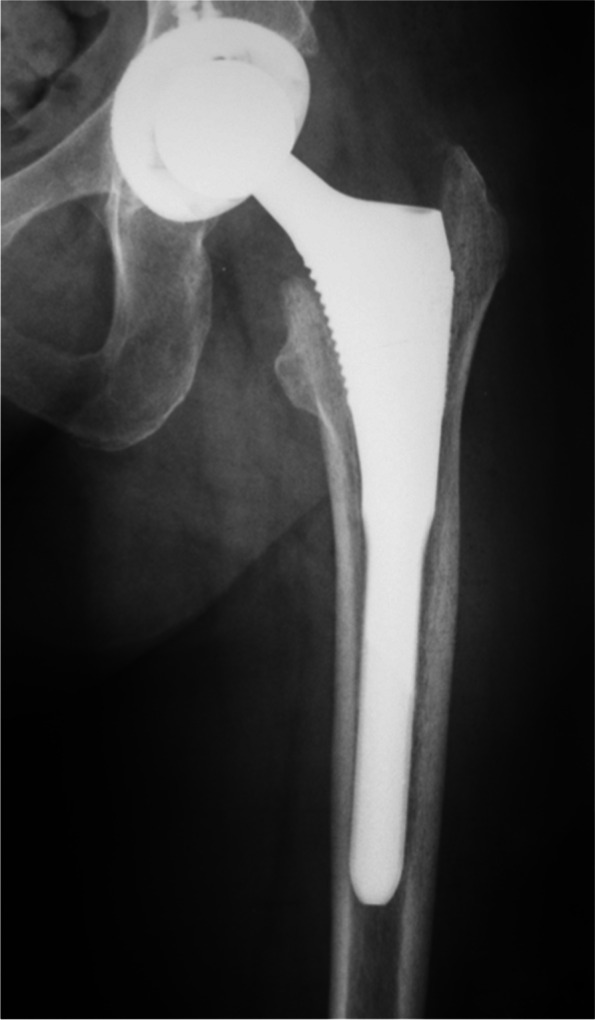
This is a representative postoperative radiograph demonstrating a well-fixed modular femoral stem

Radiographic review of the most recent films of all patients excepting the nine lost to follow-up revealed well-fixed stems with stable osseointegration in 100% of cases at an average of 60 months (range, 6 months – 16 years). There were no cases of aseptic loosening of the femoral component. There were no radiolucent lines around the coated portion of the stems, no subsidence, no lytic changes, and no pedestal formation (Fig. 4). There were no leg length discrepancies of greater than 10 mm, and 85% within 5 mm.

## Discussion

The objective of this study was to evaluate the average 15-year survivorship of a unique dual-modular femoral stem. We hypothesized that this implant would demonstrate survivorship comparable to that of a non-modular device. Our results confirmed this hypothesis, with no failures attributed to modularity of the femoral device. The authors believe that this is one of the largest studies with long term follow-up demonstrating 100% survivorship of a dual-modular femoral hip device.

Though there are unique advantages of dual modularity, numerous reports and systemic reviews have found that many modular designs have had high failure rates and recalls [8–14], and most surgeons have ceased using them in clinical practice [7]. The 2012 Australian Orthopaedic Association National Joint Replacement Registry Annual Report showed that the revision rate due to mechanical failure of modular stems compared to standard stems was 10.6% vs. 6.3% [6]. The problem became so widespread that several authors have published guidelines for evaluating and treating the potentially failed modular stem [33–35].

Failures have been especially obvious with the dual-taper exchangeable-neck designs, which share unique design weaknesses, leading to breakage [12, 17–19, 21, 22] and/or inflammation (ALTR) [15, 16, 20, 23, 36–38]. The additional modularity weakens the implant, which has been particularly problematic in obese patients [4, 8, 14, 19, 21, 23]. This has been observed primarily in the single metal couple, titanium-titanium modular neck-body designs, which may share an inadequately robust junction between the neck and stem body.

In response to the issue of implant breakage, some designs utilized a cobalt-chromium neck for increased strength, coupled with a titanium stem, which introduced mechanically-assisted crevice corrosion due to dissimilar metals, leading to ALTR [13, 38–40]. Corrosion can weaken the structural integrity of the stem, increasing risk of mechanical failure, and cause the generation of particulate debris, inducing inflammatory reactions [5, 11, 19–22, 38]. The recalled ABG II dual-taper system [23, 37, 38] as well as the Stryker Rejuvenate [15, 16, 20, 23] system have demonstrated this mode of failure.

None of our patients experienced any mechanical failure of the modular junction, breakage, or inflammation. Another report [29] has corroborated these excellent clinical results, though the first generation of this implant did exhibit breakage of the anti-rotation pin in some cases [29, 41], and others report corrosion of the pin [42]. A retrieval analysis [36] which included five cases of this stem, did not find any failure, substantial corrosion, or adverse local tissue reactions. The modular stem in this study does not have a dissimilar metal junction; both the neck and the stem are titanium alloy. The stem utilizes a “dual-press” modular connection mechanism that seats the neck fully against the proximal surface of the stem, significantly decreasing the likelihood of any gap and subsequent micromotion, allowing the assembled stem to function as a monoblock implant [29, 31, 32]. However, it is critical to fully seat the neck on the body during assembly; we have found that the original calibrated compression-limiting instrument cannot be relied upon in this regard and so utilized an alternant instrument without the compression limitation that generates a greater compressive force.

Compared to non-modular stems, dual-modular stems may allow for more anatomic restoration in leg length, hip offset, anteversion, and center of rotation, restoring each patient’s individual anatomy and biomechanics, possibly improving gait and hip stability [4, 7–12, 43–45]. Femoral offset (FO) ranges from 28 to 54 mm in the normal hip [46], and is > 45 mm in 31% of patients and > 50 mm in 12% [47]. A decrease in FO of 15% or more after THA leads to an alteration in the gait [48]. Preoperative templating of standard radiographs may underestimate FO by up to 20% on radiographs and therefore may not be restored after THA [48]. Offset was found to be restored to within 4 mm in only 25% of cases [49]. Soft tissue tension is approximately four-fold lower in recurrent dislocators and reduced FO was related to this decreased soft tissue tension [50]. Restoration of offset allows better soft-tissue balance, optimizing abductor function, reducing pain, impingement, wear and dislocation [4, 5, 13, 14].

In an effort to increase soft tissue tension, limb length may be inadvertently increased. Limb-length inequality remains a significant unsolved problem in THA [51], representing the most common reason for litigation after nerve injury, ranging from 12%-26% of lawsuits [52, 53]. Decoupling the variables of offset and length allows increased soft tissue tension through increased offset, without increased leg length [3].

While stability is influenced by both acetabular and femoral component position, the majority (65%) of dislocating THAs implanted with a posterior approach had a socket placed within the Lewinnek safe zone [54]. Another study of 7040 primary THAs revealed that this acetabular safe zone does not decrease the risk of dislocation [55], emphasizing the importance of femoral-side variables including offset, length, and version in determining hip stability. Accurate restoration of the three-dimensional geometry of the hip is crucial to hip function and implant survival [56]. Hip dislocations may increase with time, however, dislocation is usually detected in the first 3 to 6 months after surgery, with 75% occurring within the first year [57–60]. We had three patients dislocate within a year (1.7%), and one of these had revision surgery. The two early dislocations not requiring revision had head diameters ≤ 32 mm. The remainder of our dislocations (4, 2.4%) were late, and all associated with large diameter MOM hips. Overall, our dislocation rate was 4.1%, which is not low; however, a majority of these were in subjects with large diameter MOM hips with ALTR and substantial soft tissue damage. We used a conventional posterior approach without soft tissue repair of the capsule and short external rotators. Excluding the revised MOM cases, our dislocation rate was 0.6% with head size ≥ 36 mm. The incidence of dislocation after primary THA varies from 0.6% to 7% and most studies are from high-volume centers; the overall incidence in the community may be higher [61]. If instability is experienced with this device, its design allows in vivo dissassemby and modular neck exchange, allowing the surgeon to adjust offset, length and version without removing the stem itself, which has proven to be a useful though rarely required feature. The independent adjustment of offset and length is a powerful tool allowing a high intraoperative flexibility, including the ability to reduce the vertical height while simultaneously increasing offset, thus improving stability without compromising leg length.

Some argue that these potential disadvantages, plus cost considerations, and the paucity of clear-cut proven clinical advantages, limit support for the use of dual-modular stems in primary hip arthroplasty [10, 39, 62, 63]. Others support the use of dual-modular stems, when properly indicated, especially in cases of abnormal proximal femoral anatomy, avoiding obese patients with excessive offset [26–28, 30]. Modular stems have improved clinical and radiographic outcomes, including improved range of motion (ROM) and stability, in patients with abnormal proximal femoral anatomy such as dysplasia [27]. Cameron [64] has reported positively on the use of a proximally modular device, and states that the adjustability of version is beneficial. Others have reported that proximal modularity reduces hip dislocation rate, particularly in women, and that modular neck prostheses help to restore hip anatomy [30]. Modularity has been shown to significantly improve range of motion until impingment [5]. Benazzo et. al. reported a 97.5% survival at 11 years post-THA with a dual-modular stem [24].

There are limitations to our study, including its retrospective study design. Review of our records and electronic medical database did not allow us to follow-up with all patients; 9 (5%) of our patients were lost to follow-up at a mean of 4 years post-THA. Additionally, we did not perform posterior soft tissue repairs, which has been shown to decrease dislocation rate to equal that of alternative approaches [65–68]. Another limitation is that a majority of our cases used large diameter MOM, which is associated with a higher failure rate. Another weakness of our study is that we do not have metal ion levels on all subjects, this data is only available for the ten revisions performed due to MOM/ALVR-related failures.

## Conclusion

It is ill-advised to use a femoral stem that adds modularity, with the attendant potential for complications, without gaining a clear advantage. We believe the benefits may outweigh the risks, as we achieved a low early dislocation rate (1.7%) in subjects treated with a posterior approach and no posterior repair, without any leg length discrepancies; our late dislocation rate (2.4%) was in subjects who had MOM articulations with ALTR and soft tissue damage, and so these may not be attributed to the stem. The robust modular junction design mitigates the risk, and the biomechanical advantages allowing for the precise restoration of anatomy, providing maximum stability and function, without leg length discrepancy concerns, is a powerful benefit. This study demonstrates 100% long-term survivorship of a dual-modular femoral stem, an equal or higher survivorship than some non-modular stems, and a higher survival rate versus other modular femoral stems. This dual-modular device is not associated with mechanical failure or associated corrosive or inflammatory processes.

## Data Availability

All data and materials available on request from Dr. David Scott at dfscott@mac.com.
